# Extracts from *Cladiella australis*, *Clavularia viridis* and *Klyxum simplex* (Soft Corals) are Capable of Inhibiting the Growth of Human Oral Squamous Cell Carcinoma Cells

**DOI:** 10.3390/md6040595

**Published:** 2008-11-03

**Authors:** Chia-Hua Liang, Guey-Horng Wang, Chih-Chuang Liaw, Mei-Feng Lee, Shih-Hao Wang, Da-Long Cheng, Tzung-Han Chou

**Affiliations:** 1 Department of Cosmetic Science, Chia Nan University of Pharmacy and Science, Tainan, 717, Taiwan; 2 Graduate Institute of Pharmaceutical Chemistry, China Medical University, Taichung, 404, Taiwan; 3 Graduate Institute of Medicine, College of Medicine, Kaohsiung Medical University, Kaohsiung, 807, Taiwan; 4 Department of Computer and Communication, Shu-Te University, Kaohsiung County, 82445 Taiwan

**Keywords:** Soft corals, *Cladiella australis*, *Clavularia viridis*, *Klyxum simplex*, Apoptosis

## Abstract

Many biomedical products have already been obtained from marine organisms. In order to search more therapeutic drugs against cancer, this study demonstrates the cytotoxicity effects of *Cladiella australis*, *Clavularia viridis* and *Klyxum simplex* extracts on human oral squamous cell carcinoma (SCC4, SCC9 and SCC25) cells using cell adhesion and cell viability assay. The morphological alterations in SCCs cells after treatment with three extracts, such as typical nuclear condensation, nuclear fragmentation and apoptotic bodies of cells were demonstrated by Hoechst stain. Flow cytometry indicated that three extracts sensitized SCC25 cells in the G_0_/G_1_ and S-G_2_/M phases with a concomitant significantly increased sub-G_1_ fraction, indicating cell death by apoptosis. This apoptosis process was accompanied by activation of caspase-3 expression after SCC25 cells were treated with three extracts. Thereby, it is possible that extracts of *C. australis*, *C. viridis* and *K. simplex* cause apoptosis of SCCs and warrant further research investigating the possible anti-oral cancer compounds in these soft corals.

## 1. Introduction

Marine organisms are a major reservoir of bioactive natural products with potential biomedical application; several marine natural products are regarded as potential therapeutic agents for the treatment of multiple disease categories [1]. Many bioactive marine natural products and their derivatives are produced by invertebrates, such as sponges, soft corals, tunicates, mollusks or bryozoans, and are evaluated advancedly in preclinical and even clinical trials [1,2]. Moreover, from 2005 to 2007, two of 13 natural products and natural products-derived drugs approved marketing worldwide are found from marine organisms [3]. These facts attract us to pay more efforts on the research of bioactive natural products from other marine invertebrates.

Though seemingly defenseless, soft corals produce various chemical substances to discourage predation, instead of a protective calcium carbonate skeleton of hard corals as a defense mechanism. These chemicals are toxic to either predators or some neighboring hard corals, and some of these substances may have properties that can be beneficial to humans too. Because the geographic setting of Taiwan, between the West Pacific Ocean and East China Sea and at the crossroads of the Philippine-Japan I. Arc, has produced reefs of special biogeographical interest, three kinds of Formosan soft corals, including *Cladiella australis* (Alcyoniidae), *Clavularia viridis* (Clavulariidae) and *Klyxum simplex* (Alcyoniidae), were collected to be evaluated for biomedical research.

Oral cancer is a major global public health problem, causing high morbidity and mortality that have not improved in decades [4]. Squamous cell carcinomas (SCCs) are the most common type of oral cancer. Although new surgery techniques and adjuvant measures, such as chemotherapy and radiotherapy, have progressed, patients with advanced oral SCCs still have a poor prognosis, with a 5-year survival rate of 65% [5]. To develop new methods and improve existing protocols for diagnosis and treatment of SCCs become mandatory.

In the last decade, the manipulation of apoptosis (programmed cell death) has received considerable attention as a novel and promising strategy for cancer chemoprevention and therapy [6,7]. Cell apoptosis is characterized and involved by a series of typical morphological events, for example membrane blebbing, shrinkage of the cell, chromatin condensation, nuclear fragmentation, fragmentation into membrane-bound apoptotic bodies translocation of membrane phosphatidylserine and sub-G_1_ fraction, and rapid phagocytosis by neighboring cells [8]. Effective cancer therapeutic strategies often rely on preferential and efficient induction of apoptosis in tumor cells. The objective of this research was to evaluate the capability of ethyl acetate extracts of *C. australis*, *C. viridis* and *K. simplex* to inhibit the growth and induce cell apoptosis on human oral SCCs cells.

## 2. Materials and Methods

### 2.1. Tissue extraction and sample preparation

The soft corals of *C. australis*, *C. viridis* and *K. simplex* were collected *via* scuba along the coast of Southern Taiwan, at a depth of 10–15 m and were stored in a freezer until extraction. A voucher specimen was deposited at the Department of Marine Biotechnology and Resources, National Sun Yat-Sen University, Kaohsiung, Taiwan. The tissues were freeze-dried and then exhaustively extracted with ethyl acetate. The ethyl acetate extracts were then filtered and concentrated under vacuum to provide a brownish semisolid crude extract. Organic extracts were dissolved freshly in dimethylsulfoxide (DMSO) and the final concentration of DMSO did not exceed 0.1% (v/v) throughout the experiments.

### 2.2. Cell culture and treatment

Human oral SCCs cells (moderate differentiation of SCC4, poor differentiation of SCC9 and well differentiation of SCC25) were cultured in Dulbecco’s Modified Eagle Medium (DMEM)/F12 medium supplemented with 0.4 μg/ml hydrocortisone. All culture cells were purchased from American Type Culture Collection (Manassas, VA) and maintained in medium supplemented with 10% fetal bovine serum (Hazelton Product, Denver, PA, USA) and 1% penicillin-streptomycin at 37°C in 5% CO_2_ humidified atmosphere. At various concentrations after the treatment, the cells were processed for the analyses of cell adhesion, cytotoxicity, morphology changes, cell cycle, and apoptosis.

### 2.3. Measurement of cell adhesion

Cells (1.5 × 10^5^ cells/well) were subcultured into 24-well plates and incubated. After 24 h of incubation, the medium was changed by adding medium containing 1% bovine serum albumin (BSA) and with or without serial concentrations of extracts for 18 h. Attached cell number was estimated using a DNA carmine-based colorimetric method [9]. Briefly, cells were fixed with 100% methanol, dried, and stained with alcoholic/HCl carmine. Colorant was extracted with 0.01 N NaOH, and absorbance was determined at 540 nm. The cell number was estimated using a titration curve of cell density, and results were given as a percentage of the cell number with respect to control cells. For the titration curve, cells were plated at densities ranging form 1 × 10^3^ to 7 × 10^5^ cells/well in 24-well plates using serial dilutions of concentrated cell suspensions. After adhesion, some wells of each density were harvested with trypsin and cells were counted in a hemacytometer; meanwhile, parallel cultures were fixed and stained as described above [9]. A relationship between the cell number and resultant absorbance after the colorant extraction, for each cell density, was accomplished and cell-density titration-curve construction, which measured cell adhesion.

### 2.4. Determination of cell viability

Cells (1.5 × 10^4^ cells/well) were seeded in each 100 μl of 96-well multi-dishes for at least 24 h and then treatment with serial concentrations of extracts for 18 h. After replacing new medium, cellular cytotoxicity were determined by MTS [3-(4,5-di-methyl-thiazol-2-yl)-5-(3-carboxymethoxyphenyl)-2-(4-sulfophenyl)-2H-tetrazolium, inner salt] assay (CellTiter 96^™^ AQ, Promega, Madison). The absorbance at 490 nm was measured by a multi-mode microplate reader (Synergy^™^2, BioTek Instruments, Inc., USA). Values are expressed as the percentage of mean cell viability relative to the untreated cultures. The IC_50_ and IC_80_ were calculated from the drug concentration that induced 50% and 80% of cell viability rate.

### 2.5. Assessment of cell morphological changes

Cells (1.5 × 10^5^ cells/well) were plated in 24-well plates then treated with IC_50_ concentrations of extracts for 18 h. After incubation, the medium was removed and cells were fixed in 4% paraformaldehyde and permeabilized in saponin (0.1% v/v in PBS-BSA). To assess specific apoptosis, Hoechst 33342 (1 μg/ml) (Promega, USA) was added to each well and further incubated at 37°C for 30 min in the dark. Living and apoptotic cells were visualized through blue filter of fluorescence inverted microscope (Nikon, TE2000-U, Japan) at 200× magnification.

### 2.6. Assessment of cell cycle distribution and apoptotic cells

Cells (1.5 × 10^5^ cells/well) were seeded in 24-well plates and incubated with or without IC_50_ concentration of extracts for 18 h. Cells were then fixed in 70% ethanol/PBS, pelleted and resuspended in buffer containing 200 μg/ml RNase A and 0.01 mg/ml propidium iodide (PI). The cells were incubated in the dark for 15 min at room temperature and then analyzed by FACScan flow cytometer (Becton Dickinson, San Jose, CA). The cell distribution in each phase (sub-G_1_, G_0_/G_1_, S and G_2_/M phases) of the cell cycle was determined using Windows Multiple Document Interface software (WinMDI), including subG_1_-peak of apoptotic cells.

### 2.7. Determination of the activation of caspase-3 expressions

Cells were treated with IC_80_ concentration of extracts for 18 h and the expressions of caspase-3 were studied. Cells were stained with mouse anti-cleaved caspasae-3 monoclonal antibodies (1:300) (Santa Cruz, CA) in 1× PBS containing 0.5% BSA (PBS-BSA) and 0.1% sodium azide (Sigma–Aldrich) for 45 min at 4°C. Cells were then washed twice with cold PBS and incubated with FITC-conjugated anti-mouse IgG (1:500) (Santa Cruz, CA) at 4°C for 30 min. Cells were washed with cold PBS and fixed in 4% paraformaldehyde. The cell nuclei were stained with 0.1 μg/ml of Hoechst 33342 (Promega, USA) and inspected using a fluorescent microscope (Nikon, TE2000-U, Japan).

### 2.8. Quantitative analysis of the activation of caspase-3 expressions

Cells were treated with IC_50_ and IC_80_ concentration of extracts for 18 h and the expressions of caspase-3 were studied. Cells were stained with mouse anti-cleaved caspasae-3 monoclonal antibodies (1:300) (Santa Cruz, CA) in 1× PBS containing 0.5% BSA (PBS-BSA) and 0.1% sodium azide (Sigma–Aldrich) for 45 min at 4°C. Cells were then washed twice with cold PBS and incubated with FITC-conjugated anti-mouse IgG (1:500) (Santa Cruz, CA) at 4°C for 30 min. Cells were washed with cold PBS and fixed in 4% paraformaldehyde. The cell nuclei were stained with 0.1 μg/ml of Hoechst 33342 (Promega, USA). For percentage of fluorescent staining analysis, caspase-3 expressions (Ex, 495 nm; Em, 525 nm) and the cell nuclei (Ex, 346 nm; Em, 460 nm) were measured from three independent experiments by Multi-Detection Microplate Reader (Synergy^™^2, BioTek Instruments, Inc., USA) and calculated using Gene5^™^ software.

### 2.9. Statistical analysis

To evaluate the statistical significance of the difference of all the values, statistical analysis was performed on the means of the triplicates of at least three independent experiments using a two-tailed Student’s *t*-test. *P* values less than 0.05 was considered significant for all tests.

## 3. Results

### 3.1. Effect of soft corals extracts on cell adhesion

**Figure 1.**
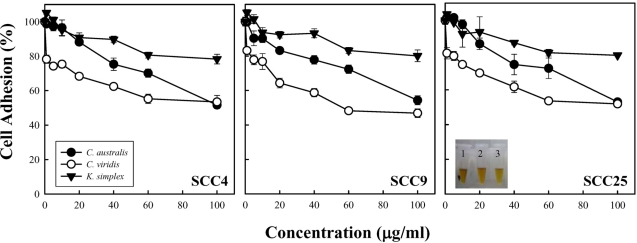
Inhibition of cell adhesion by *C. australis*, *C. viridis* and *K. simplex* extracts of SCCs cells. Percentage of viable in SCC4, SCC9 and SCC25 cells treated with serial concentrations (0–100 g/ml) of extracts for 18 h as determined by cell adhesion assay. Data are means ± S.D. from three independent experiments. *C. australis*, 1 (•); *C. viridis*, 2 (○); and *K. simplex*, 3 (▾).

To investigate the influence of extracts (*C. australis*, *C. viridis* and *K. simplex*) on cell density, constant amounts of extracts (0–100 μg/ml) were treated with SCC4, SCC9 and SCC25 cells for 18 h and then the cell adhesion was analyzed. Carmine, a natural stain is widely used for chromosome staining in cytological studies, and chromosome-specific stain method is highly accurate in a broad spectrum of cell types and cell densities [9]. As shown in Figure 1, the addition of different concentrations (0–100 μg/ml) of extracts to cancer cells for 18 h inhibited cell attachment from the culture dish surface. Although the inhibition of three cell lines generally increases with concentration, the increase is not a linear function of concentration. The *C. viridis* extract was the most potent inhibitor of both cells adhesion, which is better than those of *C. australi* and *K. simplex*. These experimental results indicate that these extracts may influence cell connection on collagen fibers, thus probably raising cell cytotoxicity. To determine further the effects of extracts on cell viability, an enzymatic tetrazolium combined with high-sensitivity test, such as a MTS assay, is necessary.

### 3.2. Effect of soft corals extracts on cells growth

In order to examine cytotoxic effects of soft corals extracts, the MTS assay were carried out using various doses of extracts on SCC4, SCC9 and SCC25 cells. As shown in Table 1, the concentrations of extracts (*C. australis*, *C. viridis* and *K. simplex*) that caused 50% cell growth inhibition (IC_50_) were approximately 39.4, 52.7 and 53.8 μg/ml for SCC4, 38.7, 31.5, 49.3 and 48.5 μg/ml for SCC9, and 38.7, 48.9 and 49.1 μg/ml for SCC25 cells, respectively. The inhibitory 80% concentration (IC_80_) of the three extracts were 95.2, 138.5 and 96.1 μg/ml for SCC4, 96.4, 127.3 and 97.6 μg/ml for SCC9, and 93.1, 132.8 and 93.3 μg/ml for SCC25, respectively.

### 3.3. Effect of soft corals extracts on cell morphological changes

**Figure 2.**
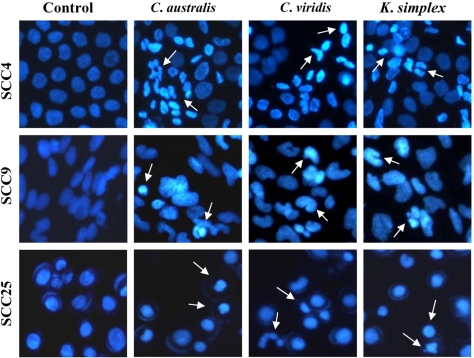
Morphological changes in SCCs cells after *C. australis*, *C. viridis* and *K. simplex* extracts treatment. The SCC4, SCC9 and SCC25 cells were seeded in 24-well plates and then treated with or without the extracts (IC_50_) for 18 h. The cells were then fixed in 4% formaldehyde and DNA stained with Hoechst. The nuclei of the cells were visualized using an inverted fluorescent microscope (200×) and photographed as described under the Methods Section.

To investigate if the viability decrease was due to a specific death type, cellular shape and nuclear morphology of exposed and *C. australis*, *C. viridis* and *K. simplex* extracts-treated SCC4, SCC9 and SCC25 cells were analyzed. As depicted in Figure 2, cells treated with soft corals extracts (IC_50_) presented morphological characteristics of apoptosis, including chromatin condensation and nuclear fragmentation, were observed under fluorescence inverted microscope. Thereby, the results could imply that three extracts may cause apoptosis of human SCCs cells.

### 3.4. Effect of soft corals extracts on cell cycle distribution and apoptosis assay

**Figure 3.**
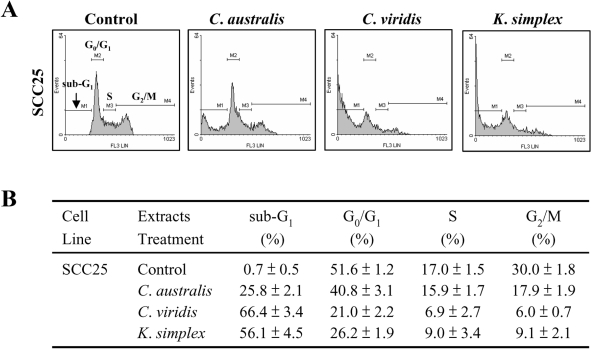
Effects of *C. australis*, *C. viridis* and *K. simplex* extracts on the cell cycles of SCC25 cells. Flow cytometric analysis of the cell cycle of SCC25 cells after three extracts (IC_50_) treatment for 18 h. (B) The percentage of cell population in the cell cycle of SCC25 cells after three extracts (IC_50_) treatments for 18 h. The cell populations were determined by WinMDI software. Data are means ± SD from three independent experiments.

To confirm that *C. australis*, *C. viridis* and *K. simplex* extracts mediate SCC25 cell apoptosis, the cell cycle distribution and specific DNA content in the sub-G_1_ peak were further investigated with flow cytometric analysis. Treatment of cells with IC_50_ concentration of the *C. australis*, *C. viridis* and *K. simplex* extracts suggests that the main characteristic of apoptosis is the cleavage of nuclear DNA into multiple fragments and causing increase in the sub-G_1_ phase (Figure 3A). Incubation with the three extracts showed a typical pattern of DNA content that reflected in the percentage of G_0_/G_1_ and S-G_2_/M phases of the cell cycle together with a marked apoptotic sub-G_1_ phase in SCC25 cells as listed in Figure 3B.

### 3.5. Effect of soft corals extracts on the expression of caspase-3

**Figure 4.**
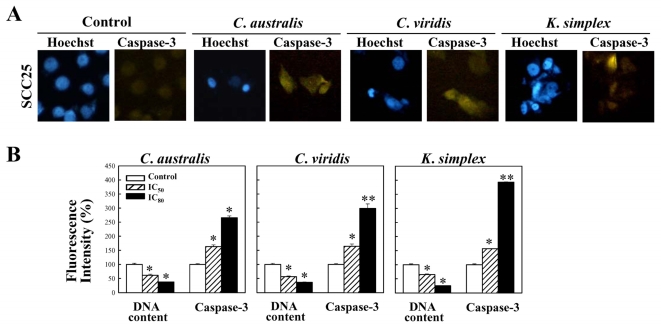
Expression of caspase-3 in SCC25 cells after treatment with *C. australis*, *C. viridis* and *K. simplex* extracts. (A) An IC_80_ concentration of extracts was added to SCC25 cells and the expression of caspase-3 were determined by immunofluorescent analysis as described in the Methods Section. (B) The IC_50_ and IC_80_ concentrations of extracts were added to SCC25 cells and the percentage of fluorescent stained caspase-3 expression levels were analyzed by Synergy^™^2 and calculated by Gene5^™^ software. Each value represents the mean ± S.D. from three independent experiments. * Indicates *P* < 0.05, ** indicates *P* < 0.01, paired Student’s *t*-test.

To determine whether caspase-3 activity are involved in SCC25 cells apoptosis induced by *C. australis*, *C. viridis* and *K. simplex* extracts, immunofluorescence analysis was performed. Up-regulation of caspase-3 expression upon exposure to IC_80_ concentration of extracts in SCC25 was obtained in Figure 4A. A significant decrease in the percentage of DNA content in SCC25 cells with concentration of IC_50_ concentration of extracts and was confirmed by cell cytotoxicity. The percentage of caspase-3 expression levels in *C. australis*, *C. viridis* and *K. simplex* extracts (IC_50_ and IC_80_)-treated SCC25 cells were around 1.6–2.7, 1.6–3.0 and 1.6–3.9 times, compared with untreated control cells (Figure 4B). Hence, these experimental results suggest that up-regulation of caspase-3 expressions by the extracts may partially account for the cell apoptosis phenomenon in SCC25 cells.

## 4. Discussion

Coral reef is a basic and important biomass in ocean. The ecological factors in coral reef, such as species competition and limiting resources in space and light, lead soft corals to develop a delicate chemical balance for self protection, range from chemical defense against predation to intra-specific cues for larval settlement. Meanwhile, some of these secondary metabolites have also shown potential as new medicines for the treatment of a variety of human diseases. During the past decades, intense attention has been focused on the anti-tumor property of marine natural products and their derivatives, such as pachymatismin from the marine sponge (*Pachymatisma johnstonii*), didemnin B from a Caribbean tunicate (*Trididemnum solidum*) and bryostatins from the bryozoan (*Bugula neritina*) [10]. Soft corals (coelenterata, octocorallia, alcyonaceae) are a rich source of steroids and terpenoids [2,11], in which most isolated diterpenes are cembranolides [12]. In addition, marine eicosanoids, such as clavulones, halogenated clavulones and prostanoids, have attracted much interest because of their novel structures and significant antitumor, antileukemic effect and antiviral activities [13]. These prostanoids have been found in the different species of marine organism such as *C. viridis*, *Telesto riisei*, and *Gracilaria* sp. [14]. Additionally, the *C. viridis* is rich in bioactive prostanoids with different structural types such as preclavulone lactones, clavirins, tricycloclavulone, and clavubicyclone [14,15]. The prostanoids have been suggested to display anti-tumor activities in several types of human tumors [14–16]. Recently, several new marine prostanoids isolated from *C. viridis* among these prostanoids, bromovulone III showed inducing of Fas clustering [17], enhancing of endoplasmic reticulum stress [10] and promising anti-tumor activity and apoptosis in human hormone-resistant prostate cancers and human hepatocellular carcinoma cells [10,17]. Previous reports have shown that eunicellin-based diterpenoids, cladiellane diterpenes and australins A-D from the ethanol extract of *C. australis* has cytotoxic activity against human breast cell lines (MCF-7, MDA-MB-231) and liver cell lines (HepaG2/DMEM-12) [18]. However, none thorough cytotoxicity research has been performed on *K. simplex* and the mechanisms underlying the anti-oral cancer efficiency of *C. australis*, *C. viridis* and *K. simplex* extracts and their secondary metabolites are poorly understood. Additional research is required to confirm the inhibitory effects and action mechanisms of *C. australis*, *C. viridis* and *K. simplex* extracts on different cancer cells and possible clinical use. This study shows the action mechanism of *C. australis*, *C. viridis* and *K. simplex* organic extracts in the human oral SCCs cells.

Cell density and cell proliferation is usually evaluated during *in vitro* studies to pharmacological effects of specific compounds [9]. In this study, cell adhesion and MTS assay revealed that *C. australis*, *C. viridis* and *K. simplex* extracts impeded SCC4, SCC9 and SCC25 cells growth in a concentration-dependent manner. The *C. viridis* was the most potent inhibitor of cells adhesion and exhibited the activity in inhibiting cell growth; that is, better than those of *C. australis* and *K. simplex*. Nevertheless, the intrinsic structure and properties of these three soft corals are still not to be clarified. Additionally, the relation yield of compounds purified form soft corals is too few to carry out apoptosis experiments. This study is a preliminary test for cytotoxic activity of soft corals and very few correlated researches could be found. At least, these results could provide the useful information to determine whether it is worthy to further isolate the natural product or not.

Although 5-fluorouracil, 5% imiquimod cream, and 3% diclofenac gel are available as agents for topical skin cancer field therapies. Nevertheless, previous reports have shown that a successful treatment of topical 5-fluorouracil cream is inevitably accompanied by pain, pruritus, burning, erythema, erosion, and scar, not only in diseased skin but also occurred in peripheral normal skin [19]. In addition, previous reports have demonstrated that clinical drugs such as 5-fluorouracil and imiquimod incubated with cancer cell lines exhibited obvious cytotoxicity after treatment extension to 72 h. The concentrations of 5-fluorouracil that induced cell death by 50% (IC_50_) were approximately 46.3 μg/ml for SCC25 cells (data not shown). Therefore, no significantly cytotoxic difference between these extracts and 5-fluorouracil could be found at the same and short interaction time for 18 h. Maybe the more deeply research is necessary in the near future to realize the true effect of these compounds on oral cancer cells.

Cancer is characterized by deregulated cell proliferation combined with suppressed apoptosis [20]. Most chemotherapeutic drugs exert their cytotoxic action by inhibiting cancer cell growth and inducing apoptosis [21]. Previous studies have demonstrated that *Sinularia* sp. extracts exhibited potent cytotoxicity against A431 cells [22] and NAKATA cells [23], and induced apoptotic DNA fragmentation and condensation of chromatin in A431 cells obtained from SCC [24]. In this study, morphologic alterations, nuclear chromatin condensation and formation of apoptotic bodies demonstrate that *C. australis*, *C. viridis* and *K. simplex* extracts cause apoptosis of SCCs cells.

Cells contain various pathways designed for protection from the genomic instability or toxicity that cell cycle play a pivotal role of this response. During tumorigenesis, tumor cells frequently loose checkpoint controls, which facilitate tumor development. Therefore, one important approach for cancer chemotherapy is to regulate cell-cycle progression. Previous reports have shown that clavulone and the halogenated analogues are anti-tumor prostanoids isolated from the *C. viridis*. It has been reported that clavulones show the antitumor activities against human leukemia HL-60 cells through the arrest of the cell cycle progression from G_1_ to S phase [25]. In this study, the cell cycle distribution indicates that *C. australis*, *C. viridis* and *K. simplex* extracts sensitized cells in the G_0_/G_1_ and S-G_2_/M phases with a concomitant significant increase in the sub-G_1_ phase. Additionally, the caspases family can be divided into major subgroups based on their substrate-specificity, sequence homology and biochemical functions; particularly, caspase-3 plays a pivotal role in the terminal phase of apoptosis [26]. This study has revealed that *C. australis*, *C. viridis* and *K. simplex* extracts may accelerate caspase-3 activation, thus resulting in apoptosis of SCC25 cells.

This study first presented evidence that human oral SCCs cells are sensitive to ethyl acetate extracts from *C. australis*, *C. viridis* and *K. simplex*. In conclusion, the present evidence suggests that *C. australis*, *C. viridis* and *K. simplex* extracts exerts its cytotoxicity effect through inhibition of the cell cycle and induction of apoptosis underwent activation of caspase-3 in SCCs cells. But the exact pathway such as what is the receptor involved in the caspase-3 activation is still unclear in these studies. Apparently, further investigations are needed for future studies.

## Figures and Tables

**Table 1.** Cell viability of *C. australis*, *C. viridis* and *K. simplex* extracts in SCCs cells.

Cell Line	Treatment	*C. australis*	*C. viridis*	*K. simplex*
SCC4	**IC**_50_	39.4 ±2.7	52.7 ± 1.6	53.8 ±2.1
	**IC**_80_	95.2 ± 1.4	138.5 ±3.9	96.1 ± 1.8
SCC9	**IC**_50_	38.7 ± 1.3	31.5 ±1.5	49.3 ±2.7
	**IC**_80_	96.4 ±2.5	127.3 ±4.1	97.6 ±3.3
SCC25	**IC**_50_	38.7 ±3.5	48.9 ±2.4	49.1 ±4.4
	**IC**_80_	93.1 ±2.1	132.8 ±4.2	93.3 ±2.9

• Results are the average of three independent experiments.

## References

[1] Radhika P, Rao PR, Archana J, Rao NK (2005). Anti-inflammatory activity of a new sphingosine derivative and cembrenoid diterpene (lobohedleolide) isolated from marine soft corals of *Sinularia crassa* TIXIER-DURIVAULT and *Lobophytum* species of the Andaman and Nicobar Islands. Biol. Pharm. Bull.

[2] Ruggieri GD (1976). Drug from the sea. Science.

[3] Butler MS (2008). Natural products to drugs: natural product-derived compounds in clinical trials. Nat. Prod. Rep.

[4] Chen W, He FY, Li YQ (2006). The apoptosis effect of hispolon from *Phellinus linteus* (Berkeley & Curtis) Teng on human epidermoid KB cells. J. Ethnopharmacol.

[5] Mork J (1998). Forty years of monitoring head and neck cancer in Norway-no good news. Anticancer Res.

[6] Lopéz L, Villavicencio MA, Albores A, Martínez M, de la Garza J, Meléndez-Zajgla J, Maldonado V (2002). Cupressus lusitanica (Cupressaceae) leaf extract induces apoptosis in cancer cells. J. Ethnopharmacol.

[7] Liang CH, Shiu LY, Chang LC, Sheu HM, Kuo KW (2007). Solamargine upregulation of Fas, downregulation of HER2, and enhancement of cytotoxicity using epirubicin in NSCLC cells. Mol. Nutr. Food Res.

[8] McCann J (1997). Texas center studies research alternative treatments. J. Natl. Cancer Inst.

[9] García-Gasca T, Paz-González V, Moncada-Alvarez MC, Blanco-Labra A, Salazar-Olivo LA (2002). Colorimetric quantitation of in vitro cell density using carmine, a chromosome-specific stain. Toxicol. In Vitro.

[10] Chiang PC, Chien CL, Pan SL, Chen WP, Teng CM, Shen YC, Guh JH (2005). Induction of endoplasmic reticulum stress and apoptosis by a marine prostanoid in human hepatocellular carcinoma. J. Hepatol.

[11] Faulkner DJ (2002). Marine natural products. Nat. Prod. Rep.

[12] Faulkner DJ (2001). Marine natural products. Nat. Prod. Rep.

[13] Iwashima M, Nara K, Iguchi K (2000). New marine steroids, yonarasterols, isolated from the okinawan soft coral, *Clavularia viridis.*. Steroids.

[14] Shen YC, Cheng YB, Lin YC, Guh JH, Teng CM, Ko CL (2004). New prostanoids with cytotoxic activity from Taiwanese octocoral *Clavularia viridis*. J Nat Prod.

[15] Watanabe K, Sekine M, Iguchi K (2003). Isolation and structures of new halogenated prostanoids from the Okinawan soft coral *Clavularia viridis*. J Nat Prod.

[16] Honda A, Yamamoto Y, Mori Y, Yamada Y, Kikuchi H (1985). Antileukemic effect of coral-prostanoids clavulones from the stolonifer *Clavularia viridis* on human myeloid leukemia (HL-60) cells. Biochem. Biophys. Res. Commun.

[17] Chiang PC, Kung FL, Huang DM, Li TK, Fan JR, Pan SL, Shen YC, Guh JH (2006). Induction of Fas clustering and apoptosis by coral prostanoid in human hormone-resistant prostate cancer cells. Eur. J. Pharmacol.

[18] Ahmed AF, Wu MH, Wang GH, Wu YC, Sheu JH (2005). Eunicellin-based diterpenoids, australins A-D, isolated from the soft coral *Cladiella australis*. J. Nat. Prod.

[19] Longley DB, Harkin DP, Johnston PG (2003). 5-fluorouracil: mechanisms of action and clinical strategies. Nat. Rev. Cancer.

[20] Yuan BZ, Jefferson AM, Millecchia L, Popescu NC, Reynolds SH (2007). Morphological changes and nuclear translocation of DLC1 tumor suppressor protein precede apoptosis in human non-small cell lung carcinoma cells. Exp Cell Res.

[21] Fribley AM, Evenchik B, Zeng Q, Park BK, Guan JY, Zhang H, Hale TJ, Soengas MS, Kaufman RJ, Wang CY (2006). Proteasome inhibitor PS-341 induces apoptosis in cisplatin-resistant squamous cell carcinoma cells by induction of Noxa. J. Biol. Chem.

[22] Fabricant RN, De Larco JE, Todaro GJ (1977). Nerve growth factor receptors on human melanoma cells in culture. Proc. Natl. Acad. Sci. U.S.A.

[23] Miyauchi S, Moroyama T, Kyoizumi S, Asakawa J, Okamoto T, Takada K (1988). Malignant tumor cell lines produce intereukin-1 like factor. In Vitro Cell. Dev. Biol.

[24] Ojika M, Islam MK, Shintani T, Zhang Y, Okamoto T, Sakagami Y (2003). Three new cytotoxic acylspermidines from the soft coral, *Sinularia* sp... Biosci. Biotechnol. Biochem.

[25] Honda A, Mori Y, Iguchi K, Yamada Y (1987). Antiproliferative and cytotoxic effects of newly discovered halogenated coral prostanoids from the Japanese stolonifer Clavularia viridis on human myeloid leukaemia cells in culture. Mol. Pharmacol.

[26] Chow KC, Lu MP, Wu MT (2006). Expression of dihydrodiol dehydrogenase plays important roles in apoptosis- and drug-resistance of A431 squamous cell carcinoma. J. Dermatol. Sci.

